# Interpretable Machine Learning for Predicting Splitting Strength of Asphalt Concrete: Insights from SHAP Analysis

**DOI:** 10.3390/ma19081636

**Published:** 2026-04-19

**Authors:** Jianglei Xing, Xiao Tan, Yihao Li, Dongzhao Jin, Pengwei Guo, Yuhuan Wang, Huiya Niu

**Affiliations:** 1 College of Water Conservancy and Hydropower Engineering, Hohai University, Nanjing 210024, China; 241602010139@hhu.edu.cn (J.X.); 2304010524@hhu.edu.cn (Y.L.); 2 State Key Laboratory of Water Disaster Prevention, Nanjing 210024, China; 3 Department of Civil, Environmental, and Geospatial Engineering, Michigan Technological University, Houghton, MI 49931, USA; 4 Faculty of Civil Engineering and Geosciences, Delft University of Technology, 2628 CN Delft, The Netherlands; p.guo-1@tudelft.nl; 5 Department of Civil, Environmental, and Architectural Engineering, University of Colorado Boulder, Boulder, CO 80309, USA; yuhuan.wang@colorado.edu; 6 Shanghai Research Institute of Building Science Co., Ltd., Shanghai 201108, China; niuhuiya@outlook.com

**Keywords:** asphalt concrete, splitting strength, TabPFN, explainable artificial intelligence, SHAP interpretation

## Abstract

This paper proposes an interpretable machine learning approach for predicting the splitting strength of asphalt concrete and supporting data-driven mixture design. A database consisting of 296 samples was constructed, and 14 input variables related to asphalt properties, aggregate gradation, and fiber characteristics were selected for modeling. Eight machine learning models, namely TabPFN, ANN, SVR, RF, XGBoost, LightGBM, FLAML, and FT-Transformer, were developed and compared. The results show that all eight models achieved satisfactory predictive capability, whereas TabPFN overall achieved the best performance in the Monte Carlo cross-validation, with the lowest average RMSE of 0.34 ± 0.10, the lowest average MAE of 0.21 ± 0.02, a relatively low average MAD of 0.14 ± 0.03, the highest average R^2^ of 0.85 ± 0.08, and the highest composite score of 0.81. SHAP analysis further indicated that splitting strength prediction was mainly governed by a limited number of dominant variables, among which Ag9.5, AC, Du, FT, and Ag4.75 were the most effective parameters. In addition, favorable parameter ranges for improving splitting strength were quantified, such as Ag9.5 < 66.8%, AC < 5.4 wt.%, Du > 134.7 cm and Ag4.75 < 45.0%. Finally, a graphic user interface platform integrating prediction and SHapley Additive exPlanations-based interpretation was developed to improve the accessibility and practical applicability of the proposed framework.

## 1. Introduction

Asphalt concrete has been extensively applied in transportation and hydraulic engineering, including road pavements [1], airport runways [2], parking areas [3], and embankment dams [4]. This wide application is mainly attributed to its favorable waterproofing ability, convenient maintenance, and economic efficiency [5,6,7]. Nevertheless, under practical service conditions, conventional asphalt concrete remains vulnerable to several problems, such as low-temperature brittleness, high-temperature deformation, and gradual deterioration caused by moisture, repeated loading, aging, and temperature variation [8,9,10]. These factors can accelerate cracking and adversely affect the durability and serviceability of pavement structures. Among commonly used mechanical performance indices, splitting strength (ST) is of critical importance because it can directly characterize the tensile resistance and crack susceptibility of asphalt concrete. Therefore, it is significant to establish reliable approaches for ST prediction and identifying major factors governing its variation for the evaluation and optimization of asphalt concrete.

Conventional experimental evaluation of the mechanical performance of asphalt concrete is generally expensive and inefficient, since changes in binder content, aggregate properties, or gradation often require repeated and time-consuming laboratory testing [11,12,13,14,15,16]. To reduce this burden, researchers have developed analytical and empirical approaches to estimate mechanical properties [17]. However, the applicability of these methods is often constrained due to simplified assumptions and limited variations even if satisfactory fitting accuracy might be achieved in specific cases. Mechanistic-based prediction frameworks have also been employed to assess long-term pavement performance, especially in relation to fatigue and rutting behavior [18]. Nevertheless, the dependence of such approaches on fixed material parameters and predefined structural assumptions reduces their generalization capability when dealing with heterogeneous asphalt mixtures [19].

Machine learning (ML) has recently become an important analytical approach in concrete materials research [20,21], particularly for identifying hidden patterns and forecasting material properties from complex datasets. Because ML methods are well suited to large, heterogeneous data and can represent nonlinear correlations among multiple material parameters, they have been increasingly adopted for investigating the mechanical performance of engineering materials [22]. In asphalt concrete, models such as artificial neural networks (ANNs), random forests (RFs), k-nearest neighbors (KNNs), and light gradient boosting machines (LightGBMs) [21,22,23,24] have already been adopted for the prediction of mechanical properties with satisfied accuracy. For example, RF achieved an R^2^ of 0.83 in predicting Marshall stability [23], while KNN and LightGBM yielded even higher predictive performance, reaching an R^2^ of 0.90 [24]. These results indicate that ML-based approaches can provide more flexible and accurate predictions than traditional empirical models, particularly when dealing with diverse mixture compositions and complex variable interactions.

To further highlight the rapid development of data-driven research in asphalt concrete, Table 1 presents an explicit comparison of representative studies published during 2022–2025. It is observed that ML has been increasingly used to predict a variety of asphalt-mixture properties, including dynamic modulus, Marshall stability, flow value, cracking resistance, stiffness modulus, and strength-related responses, with different methods, ranging from conventional regression to explainable ML models, hybrid deep learning frameworks, and application-oriented predictive tools.

Despite these recent advances, several gaps remain in the current research on asphalt concrete property prediction: (1) Explicit prediction of tensile properties of asphalt concrete, such as splitting strength, received much less attention compared with Marshall-related indicators, and existing research on predicting tensile properties of asphalt concrete is rare according to literature review. (2) Although ML methods showed strong predictive capability, many ML models lack interpretability as model complexity increases, known as the “black-box” effect [21]. It is still urgent to develop an integrated framework that combines recent models, robust comparative evaluation, and interpretable analysis. (3) Practical and publicly accessible platforms for asphalt concrete ST prediction remain scarce, which restricts the direct usability of these methods for engineers and researchers.

To address these issues, an interpretable ML framework was developed in this study for predicting the ST of asphalt concrete. A literature-derived dataset incorporating asphalt-related properties, aggregate gradation parameters, and mixture design variables was used to benchmark eight ML models, namely TabPFN, ANN, SVR, RF, XGBoost, LightGBM, FLAML, and FT-Transformer, and then the optimal model was selected through a comprehensive performance assessment. To provide insight into the prediction process, SHapley Additive exPlanations (SHAP) was employed to determine the most influential variables and to characterize how they contributed to ST variation. In addition, a graphical user interface (GUI) was built to combine prediction with interpretation, thereby facilitating practical use of the proposed method.

The novelties of this study can be summarized in three aspects: (1) Tabular Prior-data Fitted Network (TabPFN) was introduced for ST prediction of asphalt concrete because its pretrained tabular foundation architecture is well suited to the present task [32]. The ST dataset used in this study contains 296 samples and 14 heterogeneous descriptors related to asphalt properties, aggregate gradation, and fiber characteristics. For such a small-to-medium-sized tabular regression problem, TabPFN is able to leverage transferable knowledge acquired during prior training on a large number of synthetic tabular tasks, meanwhile requiring little dataset-specific tuning. (2) An interpretable ML framework was established for asphalt concrete ST prediction by combining data preprocessing, multi-model comparison, composite indicator-based evaluation, and SHAP analysis, which not only improved predictive reliability but also revealed the relative importance of input variables and their influence patterns on ST. (3) An intuitive GUI platform was developed to integrate prediction and interpretation into an interactive tool for asphalt concrete evaluation and design.

## 2. Methodology

**Figure 1:**
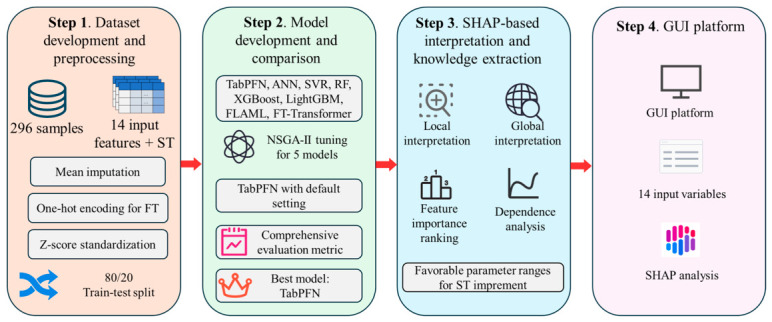
Interpretable ML framework for asphalt concrete splitting strength prediction, SHAP-based interpretation, and GUI application.

Figure 1 illustrates the workflow of the proposed interpretable ML framework, consisting of four steps:

(1) Dataset development and preprocessing, where 296 samples with 14 input variables and ST as the output were prepared using mean imputation, one-hot encoding, and Z-score standardization, followed by repeated model evaluation over 20 Monte Carlo random splits with an 80:20 training–testing ratio. (2) Model development and comparison, where eight models were developed, with NSGA-II used for hyperparameter optimization, and TabPFN identified as the best performer. (3) SHAP-based interpretation for feature importance and parameter analysis. (4) A GUI platform integrating trained model and SHAP results for interactive prediction and explanation.

All computational procedures in this study were implemented in Python 3.13.12. The main libraries covered data preprocessing, ML model development, hyperparameter optimization, SHAP-based interpretation, and GUI implementation. For reproducibility, the main software environment and library versions used in this study are summarized in Appendix A, Table A1.

### 2.1. Database Development

#### 2.1.1. Database Construction and Description

A database containing 296 asphalt concrete samples was developed from relevant studies published between 2008 and 2025 for ST prediction [33,34,35,36,37,38,39,40,41,42,43,44,45,46,47,48,49,50,51,52,53,54,55,56,57,58,59,60,61,62,63]. Taking into account the material properties of asphalt, aggregate gradation, and fibers, fourteen input variables were selected and classified into three groups to investigate their effects on splitting strength [64]: asphalt-related features, including asphalt content (AC), penetration (Pe), softening point (SP) and ductility (Du); aggregate-related features, including the passing percentages of 2.36 mm, 4.75 mm, and 9.5 mm aggregates (Ag2.36, Ag4.75, and Ag9.5), air voids (AVs), voids in mineral aggregate (VMAs), and voids filled with asphalt (VFAs); and fiber-related features, namely fiber content (FC), fiber type (FT), tensile strength (TS) and fiber length (FL). When the samples are classified according to fiber type, the database includes basalt fiber, glass fiber, polyester fiber, steel fiber, and no fiber, and their distribution proportions are shown in Table 2.

#### 2.1.2. Data Analysis

The missing values in the database were filled using mean imputation. Following imputation, the statistical profiles of all variables are summarized in Table 3, covering the minimum, maximum, quartile values, mean, and standard deviation for each variable.

**Figure 2:**
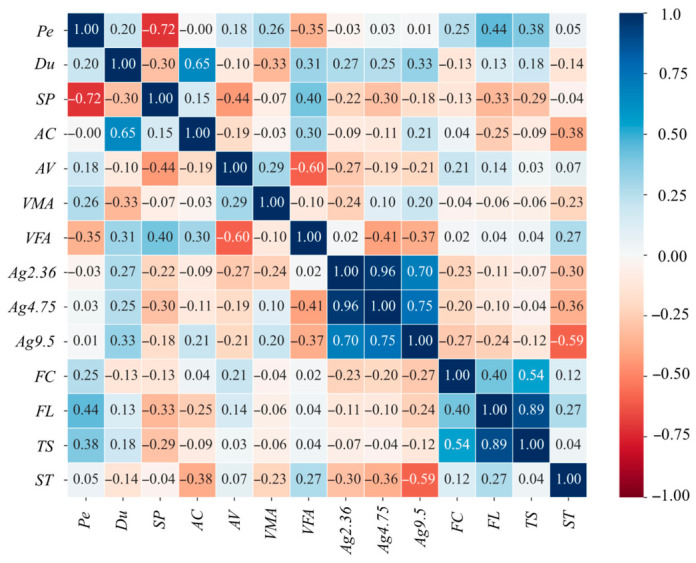
Pearson correlation heatmap of dataset.

**Figure A1:**
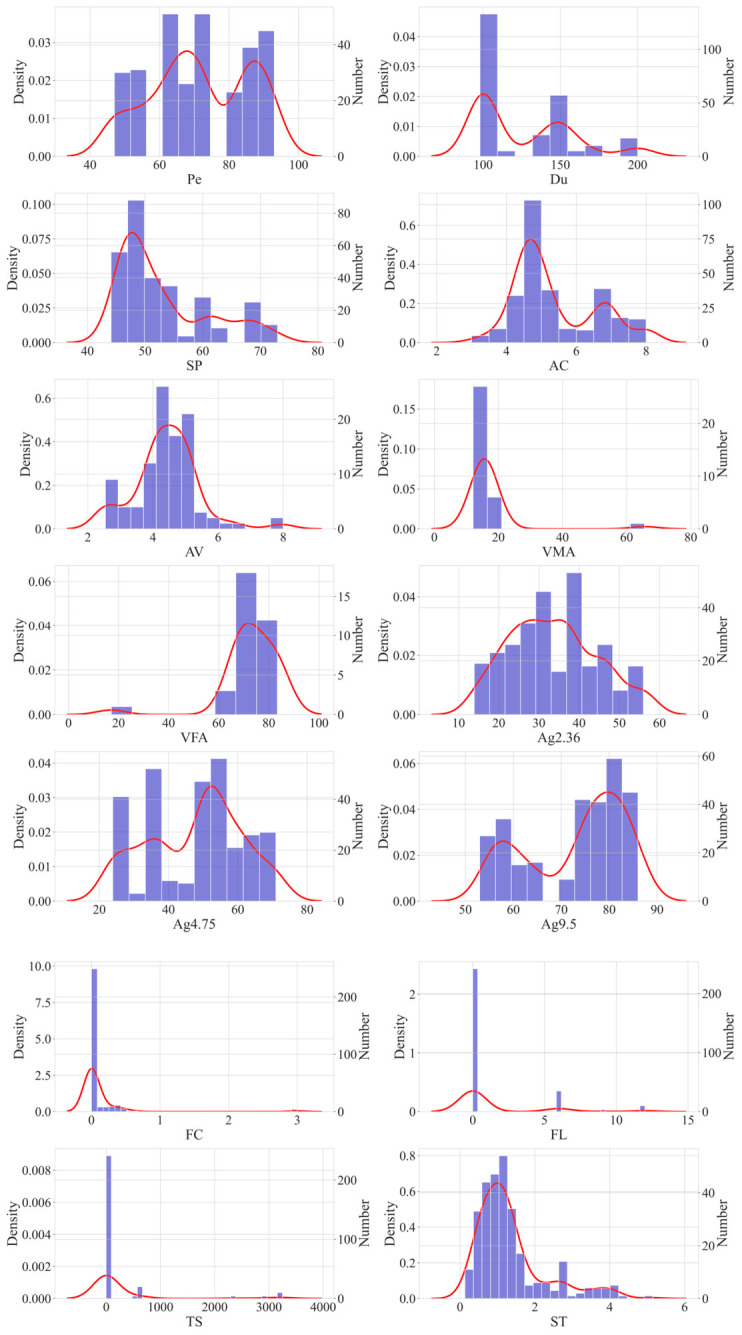
Distribution of fourteen input features and one output feature of the dataset.

The probability density characteristics of fourteen input variables and the output variable are presented in Figure A1. For each feature (such as Pe, Du, and SP), a dual-axis subplot is used, with probability density shown on the left *y*-axis and frequency shown on the right *y*-axis. Pearson correlation analysis was further carried out to evaluate possible multicollinearity, and the results are provided in Figure 2 [65]. The results show that, except for a few relatively strong correlations among asphalt, aggregate, and fiber internal features, the correlations among the remaining features all satisfy |R| < 0.7 [66]. This indicates weak linear relationships and limited redundancy among the variables, confirming that the selected features were appropriate for model training. Because no severe multicollinearity was observed among the selected variables, all mixture design variables were kept in the input set [67].

**Figure 3:**
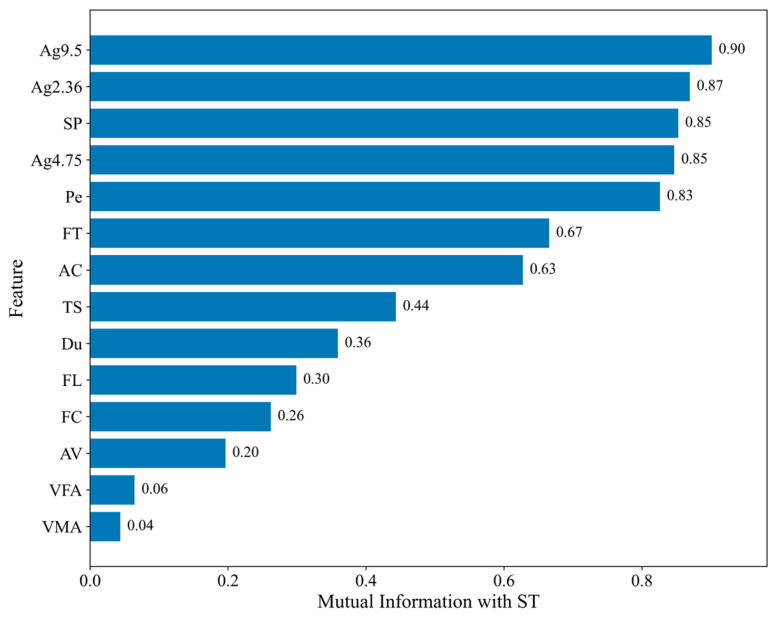
Mutual information ranking of the input variables with respect to ST.

While Pearson correlation mainly reflects linear relationships among variables, mutual information (MI) ranking was further performed to evaluate the nonlinear dependency between each input variable and ST, as shown in Figure 3. MI is able to capture more general nonlinear associations between individual features and the target variable. The results show that Ag9.5, Ag2.36, SP, Ag4.75, and Pe have the highest MI scores, followed by FT and AC, indicating that these variables are more strongly associated with ST from a nonlinear information perspective. In contrast, VFA and VMA show relatively small MI values. These findings suggest that the dependence of ST on the selected variables is not purely linear, thereby supporting the application of ML models for ST prediction.

#### 2.1.3. Data Preprocessing

For the categorical variable FT, one-hot encoding was used to make it compatible with machine learning models [68]. Numerical features were standardized using Z-score scaling before training to ensure a common scale, improving efficiency and convergence by reducing the influence of large-magnitude variables [69,70]. The formula is given as follows:

(1) $$z=\frac{x-m}{\sigma }$$

where x denotes an input variable, while m and σ represent its mean and standard deviation, respectively.

The standardized dataset was then randomly reordered to minimize possible sequence-related effects and to enhance the representativeness of the training samples [67]. After that, the dataset was repeatedly divided into training and testing subsets using 20 Monte Carlo random splits with a training–testing ratio of 80:20 [71].

### 2.2. Machine Learning Models

Eight machine learning models were employed in this study, including Tabular Prior-data Fitted Network (TabPFN) [32], Support Vector Regression (SVR) [72], Random Forest (RF) [73], Extreme Gradient Boosting Trees (XGBoost) [74], Light Gradient Boosting Machine (LightGBM) [75], Artificial Neural Network (ANN) [76], Fast and Lightweight AutoML (FLAML) [77], and FT-Transformer [78]. The classification of these models is presented in Table 4.

**Figure 4:**
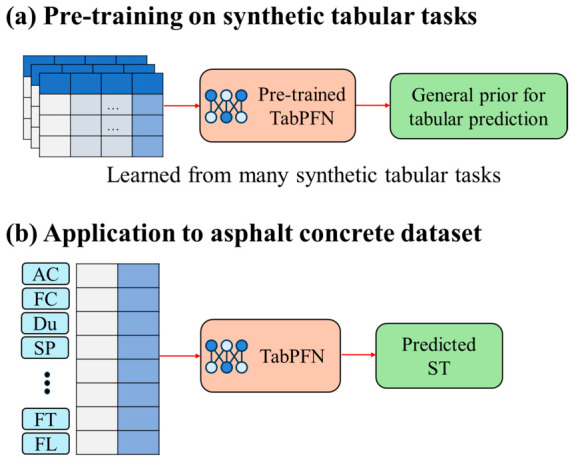
Schematic diagram of TabPFN pretraining and its application to ST prediction.

Among the eight models, TabPFN deserves particular attention because it fundamentally differs from the other models in training paradigm. As illustrated in Figure 4a, TabPFN is a pretrained tabular foundation model which is trained on a large collection of synthetic tabular tasks generated from diverse data-generating processes [79]. Through prior training, it learns general priors for tabular prediction and therefore captures transferable patterns instead of being trained entirely from scratch on present dataset. In this study, fourteen input descriptors of asphalt concrete, including asphalt-related, aggregate-related, and fiber-related variables, were organized into tabular inputs and then utilizing pretrained TabPFN model for predicting the target variable ST, as shown in Figure 4b. The underlying architecture of TabPFN is based on Transformer, in which tabular features are encoded and processed through stacked Transformer blocks before being passed to prediction head [32]. This design makes TabPFN particularly suitable for present ST database which contains a moderate number of samples and a compact set of descriptors, because TabPFN reduces the need for extensive dataset-specific tuning while still maintaining strong predictive performance. In this study, TabPFN developed by the open-source PriorLabs was used via its regressor interface for prediction [80].

The remaining seven models were introduced as representative benchmarks from classical machine learning, ensemble learning, AutoML, and transformer-based tabular deep learning.

### 2.3. Evaluation Metrics

Model performance was evaluated using five indicators: root mean square error (RMSE), mean absolute percentage error (MAPE), mean absolute error (MAE), median absolute deviation (MAD) of the residuals, and the coefficient of determination (R^2^). Their mathematical definitions are given in Equations (2)–(6). Among them, MAE reflects the average error in original units and is easily interpretable. RMSE emphasizes larger errors, while MAPE expresses error as a percentage. MAD is more robust to outliers, and R^2^ indicates the proportion of variance explained, with values closer to 1 showing better fit. In general, lower RMSE, MAPE, MAE, and MAD values indicate higher predictive accuracy, whereas a higher R^2^ suggests superior model performance.

(2) $$RMSE=\sqrt{\frac{1}{n}\sum_{i=1}^{n}(y_{i,pre}-y_{i,test}{)}^{2}}$$

(3) $$MAPE=\frac{1}{n}\sum_{i=1}^{n}\left.\left|\frac{y_{i,pre}-y_{i,test}}{y_{i,test}}\right.\right|\times 100\%$$

(4) $$MAE=\frac{1}{n}\sum_{i=1}^{n}\left.\left|y_{i,pre}-y_{i,test}\right.\right|$$

(5) $$\left\{\begin{array}{c} e_{i}=y_{i}-{\hat{y}}_{i}\\ MAD=median\left(\left|e_{i}-median(e)\right|\right) \end{array}\right.$$

(6) $$R^{2}=1-\frac{\sum_{i=1}^{n}(y_{i,test}-y_{i,pre}{)}^{2}}{\sum_{i=1}^{n}(y_{i,test}-\bar{y}{)}^{2}}$$

Note: For each target variable, n represents the number of samples in the testing (or training) set; $y_{i}$ and ${\hat{y}}_{i}$ are the actual and predicted values of the i-th sample, respectively; $\bar{y}$ is the mean of the ground-truth values; and $e_{i}$ represents the residual for the i-th sample.

Because different evaluation metrics emphasize different aspects of predictive performance, relying on a single indicator may lead to a partial assessment of model behavior. Therefore, to integrate five evaluation metrics into a unified form and enable a more intuitive comparison of model performance, the metrics were further transformed into dimensionless bounded scores within the interval (0, 1), where a larger value consistently indicates better performance. Equal weights were assigned to the five normalized metrics because no universally accepted priority weighting scheme is available for ST prediction, and this treatment avoids introducing additional subjective preference toward any single metric. The sigmoid transformation was further used to reduce the dominance of extreme standardized values and improve the stability of the aggregated comparison.

(7) $$u_{k,m}=\left\{\begin{array}{c} -x_{k,m},\;\;\;k\in \{RMSE,\;MAPE,\;MAE,\;MAD\}\\ x_{k,m},\;\;\;k\in R^{2} \end{array}\right.$$

where $x_{k,m}$ denotes the raw value of metric k for model m, and $u_{k,m}$ is the corresponding utility value after directional unification.

Then, for each metric k, z-score standardization was performed across all candidate models:

(8) $$z_{k,m}=\frac{u_{k,m}-\mu_{k}}{\sigma_{k}}$$

where $\mu_{k}$ and $\sigma_{k}$ are the mean and standard deviation of the utility values of metric k across all models, respectively.

Finally, to obtain a stable bounded score and reduce the dominance of extreme values, the standardized values were mapped into the interval (0, 1) using a sigmoid function:

(9) $$s_{k,m}=\frac{1}{1+{exp}^{\left(-z_{k,m}/\alpha \right)}}$$

where α is a scaling parameter controlling the steepness of the transformation. In this study, α = 0.5.

Based on the five normalized metric scores, the composite score of models m was calculated as the arithmetic mean of the normalized scores over the five metrics:

(10) $$Composite{\;Score}_{m}=\frac{1}{5}\sum_{k\in K}s_{k,m}$$

(11) $$\mathrm{where}\;K=\{RMSE,MAPE,MAE,MAD,R^{2}\}$$

A larger composite score indicates better overall predictive performance after jointly considering prediction accuracy, goodness of fit, and residual stability. In this study, the normalized metric scores were used to construct the radar chart, while the composite score was used to rank the overall performance of different models.

### 2.4. Hyperparameter Tuning Through Objective Optimization

Unlike conventional machine learning models, TabPFN was not subjected to hyperparameter tuning in this study. This is because TabPFN is designed as a pretrained tabular foundation model, whose predictive capability mainly stems from large-scale prior pretraining rather than dataset-specific parameter adjustment. According to the characteristics of the model itself and the recommendation of its original authors, TabPFN is intended to be used largely in its default configuration, thereby avoiding the expensive and often unnecessary hyperparameter optimization process required by many traditional machine learning algorithms. This property is also one of the practical advantages of TabPFN, especially for small-to-medium-sized tabular datasets, as it allows strong predictive performance to be achieved with minimal manual intervention [32]. At the same time, it should be noted that TabPFN benefits from prior training on synthetic tasks, which may provide an advantage over models trained solely on the present dataset. Therefore, its superior performance in this study should be interpreted as reflecting both its architectural suitability for the current ST prediction problem and the benefit of prior pretraining, rather than model novelty alone.

Similarly, FLAML and FT-Transformer are not further optimized by the external NSGA-II procedure. FLAML is based on an AutoML framework that already performs automated model selection and internal configuration search under a predefined optimization budget, so applying an additional NSGA-II search would introduce methodological redundancy. FT-Transformer is used as a transformer-based tabular benchmark to compare with TabPFN. Since both FT-Transformer and TabPFN are based on the Transformer architecture, FT-Transformer is evaluated under a fixed standard configuration rather than being further optimized, so that it could serve as a direct reference model in comparative analysis.

**Figure 5:**
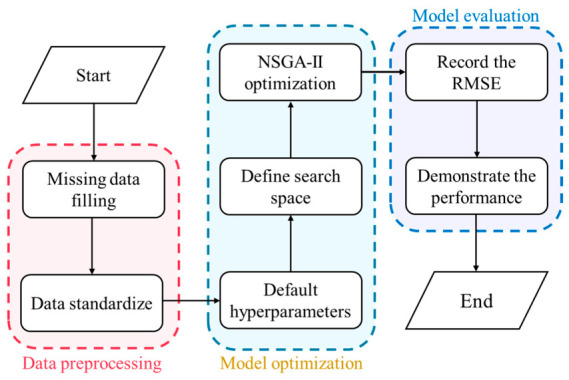
Flowchart of NSGA-II-based hyperparameter tuning process.

For the remaining machine learning models, hyperparameter optimization plays an important role in improving predictive performance. Common approaches include Grid Search [81], Random Search [82], and genetic algorithms-based method [83]. Grid Search and Random Search were not adopted in this study because they become computationally inefficient when exploring high-dimensional hyperparameter spaces and are less effective in capturing complex interactions among hyperparameters. Therefore, Non-dominated Sorting Genetic Algorithm II (NSGA-II), a Genetic Algorithm-based method, was employed. NSGA-II provides an efficient global search strategy by maintaining population diversity and balancing exploration and exploitation during the optimization process [84]. It searches hyperparameter space through an evolutionary process in which each generation contains multiple candidate solutions, and each individual represents a specific hyperparameter setting. In the present work, the tuning process aimed to minimize RMSE of target variable ST. The corresponding tuning procedure is illustrated in Figure 5.

### 2.5. SHAP-Based Model Explanation

**Figure 6:**
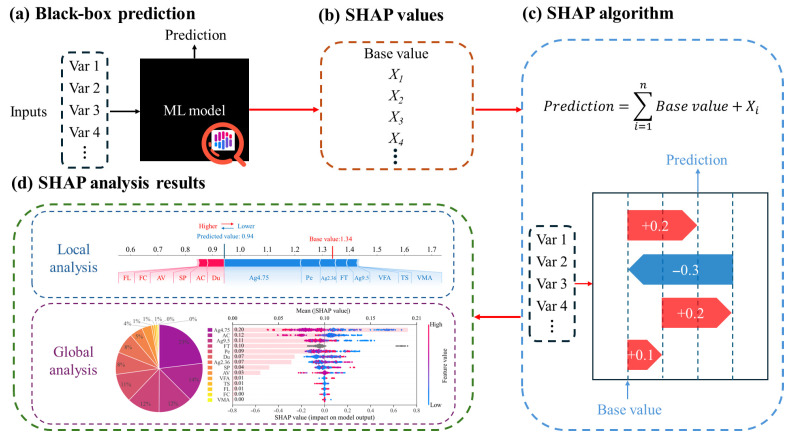
SHAP-based interpretation process for machine learning model predictions.

ML models have shown strong predictive capability in materials engineering, yet their practical application is often constrained by limited interpretability because many of them function as “black-box” systems. To address this issue, SHAP [85] can be employed as a post hoc interpretation tool to quantify the contribution of each input variable and thus provide transparent explanations for model outputs. As illustrated in Figure 6, the SHAP-based explanation framework can be understood in four parts. In Figure 6a, the input variables are first fed into the trained ML model, which operates as a black-box predictor and generates the target prediction. Figure 6b then shows that this prediction can be decomposed into a baseline value together with the contribution of individual input features, expressed as SHAP values. Figure 6c further illustrates the additive principle of SHAP, in which the final prediction is obtained by adjusting the base value through the positive or negative effects of different variables. In this process, positive SHAP values increase the prediction, whereas negative SHAP values decrease it. Finally, Figure 6d presents the interpretation results at both local and global levels. Local analysis explains how a single prediction is formed, while global analysis summarizes the overall importance and influence patterns of features across the entire dataset. In this way, SHAP converts the original black-box prediction into an interpretable explanation framework, thereby improving the transparency of the model decision-making process.

## 3. Results and Discussion

### 3.1. Hyperparameter Optimization Results

Optimal hyperparameter combinations were identified using the pymoo-based NSGA-II algorithm, which was executed with a population size of 50 over 100 generations. For each candidate hyperparameter set, the objective value was defined as the mean RMSE from 5-fold cross-validation on the training data, and the optimization process aimed to minimize this value.

**Figure 7:**
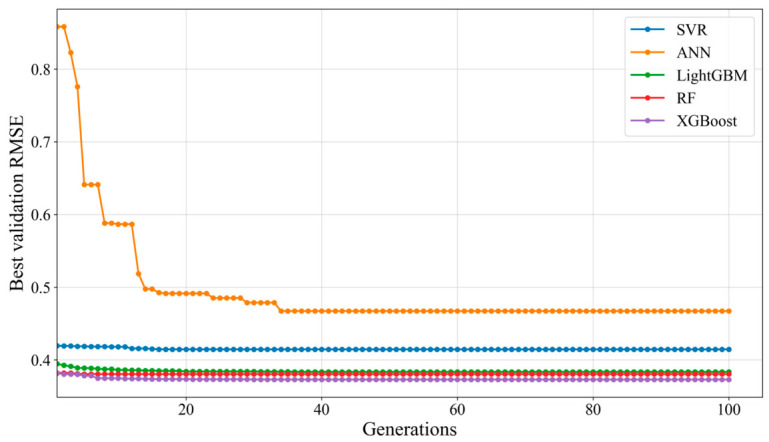
RMSE variation of models during NSGA-II iterations.

Based on the above optimization settings, the termination criterion was defined as 100 generations. As shown in Figure 7, all five models converged well before the preset maximum generation, indicating that this setting provided sufficient search depth while avoiding unnecessary computational expense. Specifically, RF, XGBoost, and LightGBM reached stable validation RMSE values within the early generations, whereas SVR showed only slight improvement after its initial convergence. By contrast, ANN exhibited a relatively slower optimization process, with a pronounced reduction in RMSE during the early generations and a gradual plateau after approximately 40 generations, followed by only marginal improvement thereafter. Overall, Figure 7 illustrates the evolution of the best validation RMSE during the NSGA-II optimization process, confirming that the selected generation limit was adequate and that extending the search further would be unlikely to produce substantial performance gains. The resulting hyperparameter combinations for all tuned models are provided in Appendix B, Table A2.

### 3.2. Prediction Performance

**Figure 8:**
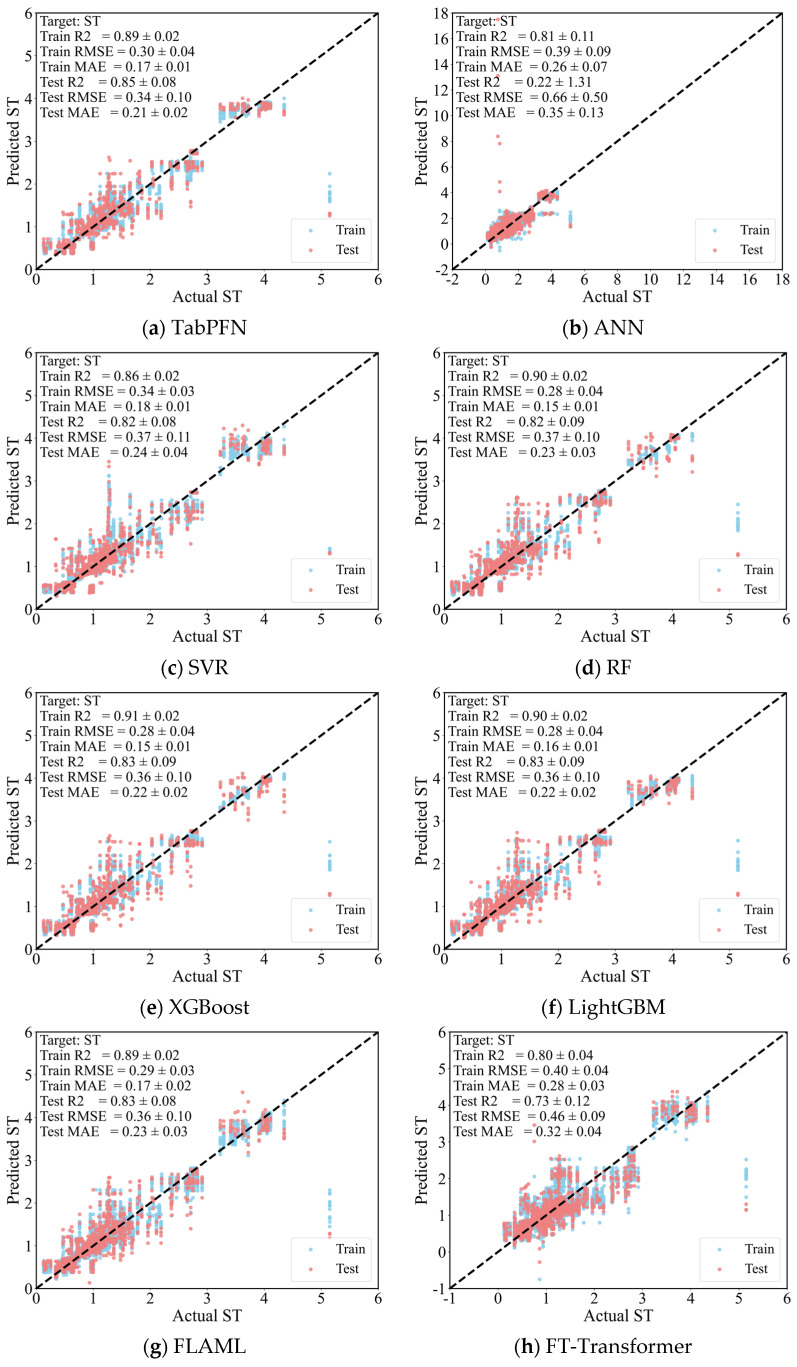
Predictive performance of eight models for ST prediction over 20 Monte Carlo random splits (mean ± standard deviation).

Figure 8 summarizes the prediction performances of the eight models over 20 Monte Carlo random splits to provide a more robust assessment of model performance than a single train–test split, expressed as the mean ± standard deviation for each evaluation metrics. This repeated random-splitting strategy reflects not only the average predictive accuracy but also the stability of each model under different data partitions. The scatter points for the two datasets are plotted against the 1:1 reference line, which represents perfect agreement between model outputs and measured values. Data points located closer to this reference line indicate stronger predictive consistency and smaller deviations.

As shown in Figure 8 and Table A3, all models were able to predict ST, but their average accuracy and robustness differed noticeably across the 20 Monte Carlo random splits. It is also noted that MAE is lower than RMSE for all models, which is consistent with the general expectation for satisfactory machine-learning predictions [86]. Overall, TabPFN achieved the best performance with the lowest average RMSE (0.34 ± 0.10), the lowest average MAE (0.21 ± 0.02), a relatively low average MAD (0.14 ± 0.03), and the highest average R^2^ (0.85 ± 0.08). XGBoost, LightGBM, RF, FLAML, and SVR formed the second-performance tier, with relatively similar mean errors and R^2^ values. By contrast, FT-Transformer exhibited weaker overall performance, and especially ANN showed the largest fluctuation across repeated random splits, as reflected by its relatively large standard deviations for RMSE and R^2^. These results indicate that TabPFN not only achieved the highest average predictive accuracy, but also maintained comparatively stability under different data partitions.

**Figure 9:**
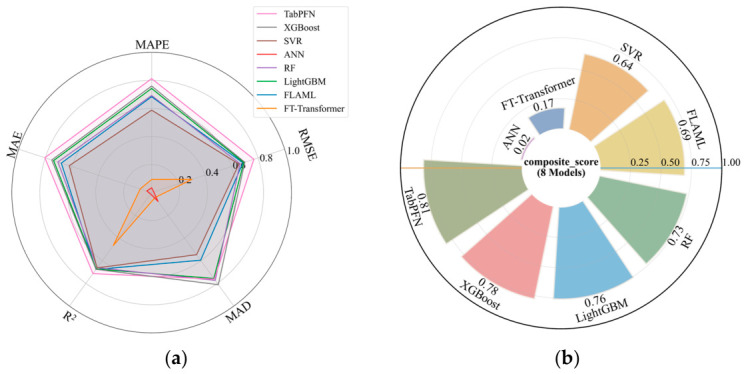
Performance evaluation of eight models for ST prediction based on Monte Carlo cross-validation results: (**a**) radar chart based on five normalized evaluation metrics; (**b**) composite score integrating five metrics.

The multi-metric predictive performance of the eight models for ST is summarized in Figure 9 based on the Monte Carlo cross-validation results. As shown in Figure 9a, TabPFN achieved the best overall performance across five evaluation metrics, showing clear advantages in RMSE, MAPE, MAE, MAD, and R^2^ after normalization. XGBoost and LightGBM ranked behind TabPFN and also exhibited relatively balanced predictive capability, while RF and FLAML showed similar overall performance. SVR performed at a moderate level, whereas FT-Transformer and ANN obtained comparatively lower scores, indicating weaker overall predictive capability under repeated random splits. To provide a more intuitive overall comparison among five evaluation metrics, the composite score was further used as a unified indicator for cross-model evaluation. As shown in Figure 9b, TabPFN reached the highest composite score of 0.81, followed by XGBoost (0.78) and LightGBM (0.76). Overall, the results indicate that TabPFN is the most effective model for ST prediction in the present dataset, while XGBoost, LightGBM, RF, and FLAML also provide relatively competitive performance.

To further examine whether the performance differences between TabPFN and the competing models were statistically significant, paired t-tests were performed on the RMSE and R^2^ values obtained from the 20 Monte Carlo random splits, as summarized in Table A4 and Table A5. TabPFN showed significant differences from the rest models in both RMSE and R^2^ after multiple-comparison correction (adjusted *p* < 0.05). The advantage of TabPFN was especially obvious when compared with ANN and FT-Transformer, while the superiority of TabPFN was relatively smaller, compared to RF, XGBoost, LightGBM, FLAML, and SVR. Overall, the statistical test results further support the robustness of superior prediction performance achieved by TabPFN.

### 3.3. Local Interpretability Based on SHAP

**Figure 10:**
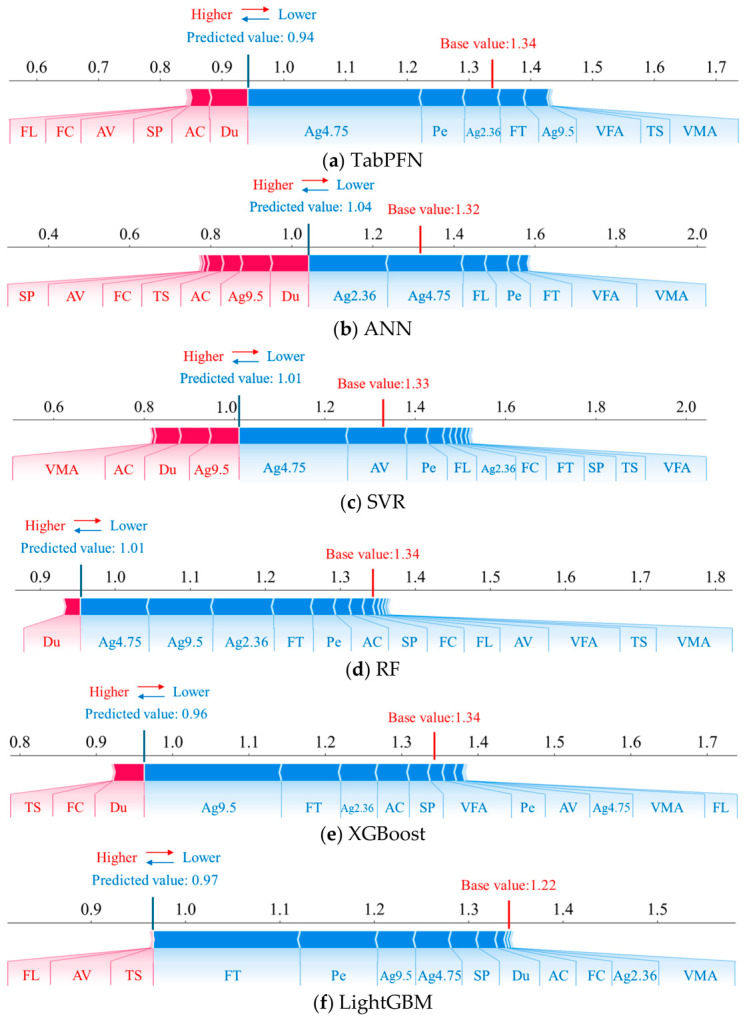

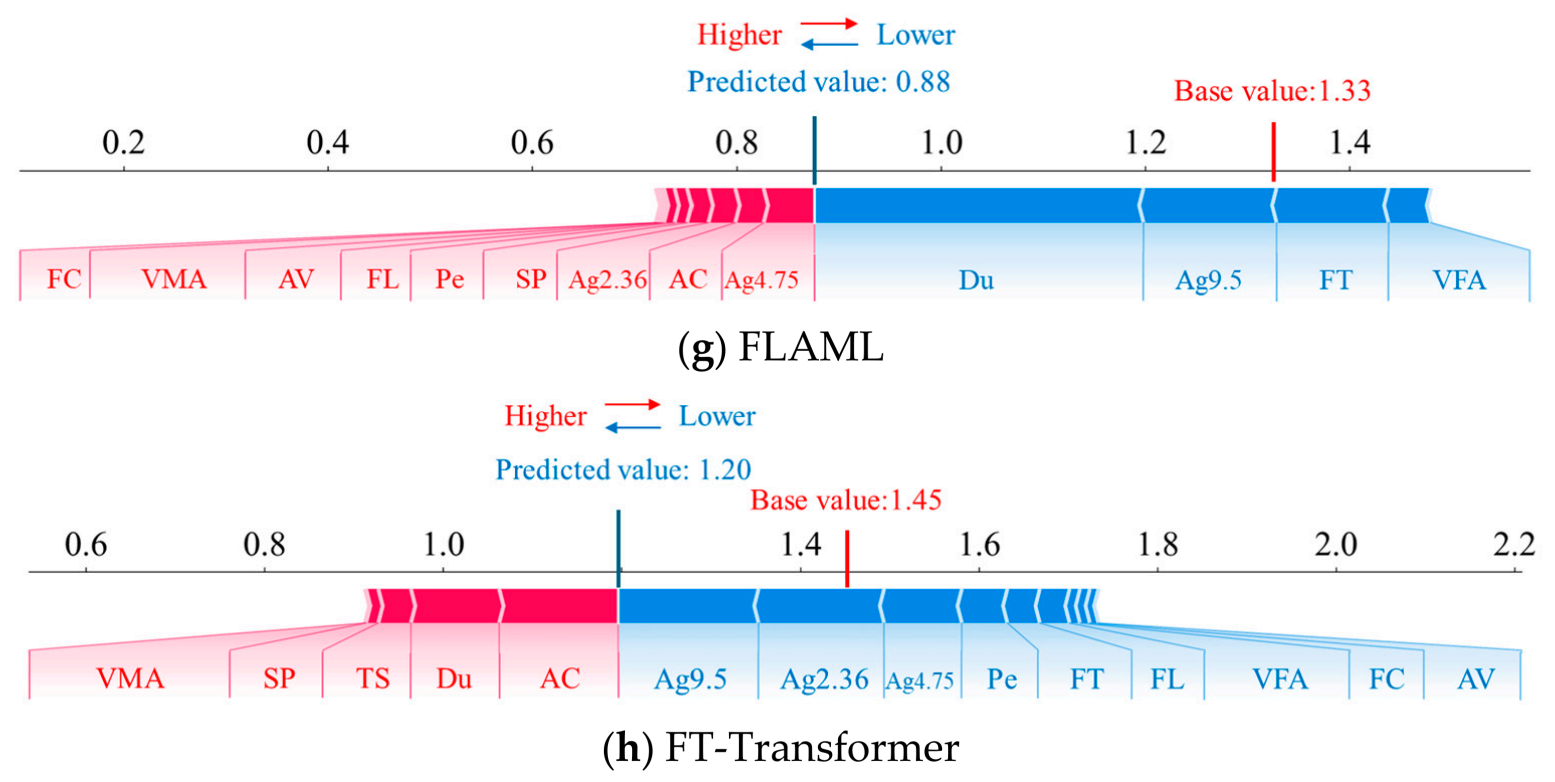
An illustration of the prediction behavior of the eight models. Red bars represent positive effects, whereas blue bars represent negative effects.

To provide a case-level interpretation of the prediction behavior of the eight models, a randomly chosen sample was examined in detail. The corresponding feature values were AC = 4.6 wt.%, Pe = 72 (0.1 mm), Du = 137 cm, SP = 51 °C, AV = 4.48%, VMA = 17.51%, VFA = 71.97%, Ag2.36 = 56%, Ag4.75 = 69%, Ag9.5 = 86%, FT = No_fiber, FC = 0 wt.%, FL = 0 mm, TS = 0 MPa, and ST = 0.93 MPa. As shown in the SHAP force plots in Figure 10, all eight models predict the selected sample at values lower than their corresponding base values, indicating an overall downward shift from the average prediction. Although the dominant contributing features vary across models, some consistent patterns can still be observed. In particular, FT (No_fiber) shows a negative contribution in all eight models, while VMA, Ag4.75, Ag2.36, and Pe also tend to reduce the predicted ST in most cases.

The final ST values predicted by TabPFN, ANN, SVR, RF, XGBoost, LightGBM, FLAML, and FT-Transformer were 0.94, 1.04, 1.01, 0.96, 0.97, 0.97, 0.88, and 1.20 MPa, respectively. Given that the actual ST value of the selected sample is 0.93 MPa, the corresponding relative errors are 1.08%, 11.83%, 8.60%, 3.23%, 4.30%, 4.30%, 5.38%, and 29.03%, respectively. Among the eight models, TabPFN yields the closest prediction to the actual value, while RF, XGBoost, LightGBM, and FLAML also maintain relatively small prediction errors for this sample. By comparison, FT-Transformer shows a markedly larger deviation, indicating that its local prediction for this case is less accurate than those of the other competitive models.

### 3.4. Global Interpretability Based on SHAP

#### 3.4.1. Contribution of Individual Features

**Figure 11:**
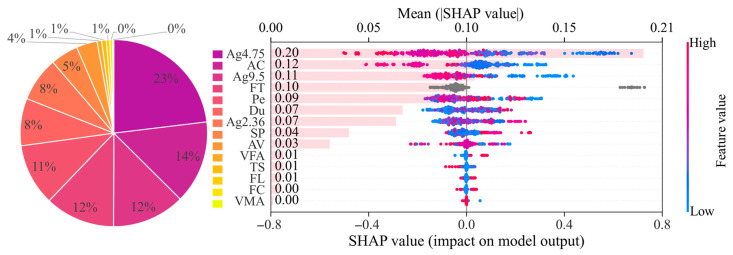
SHAP analysis of the TabPFN predictions.

**Figure A2:**
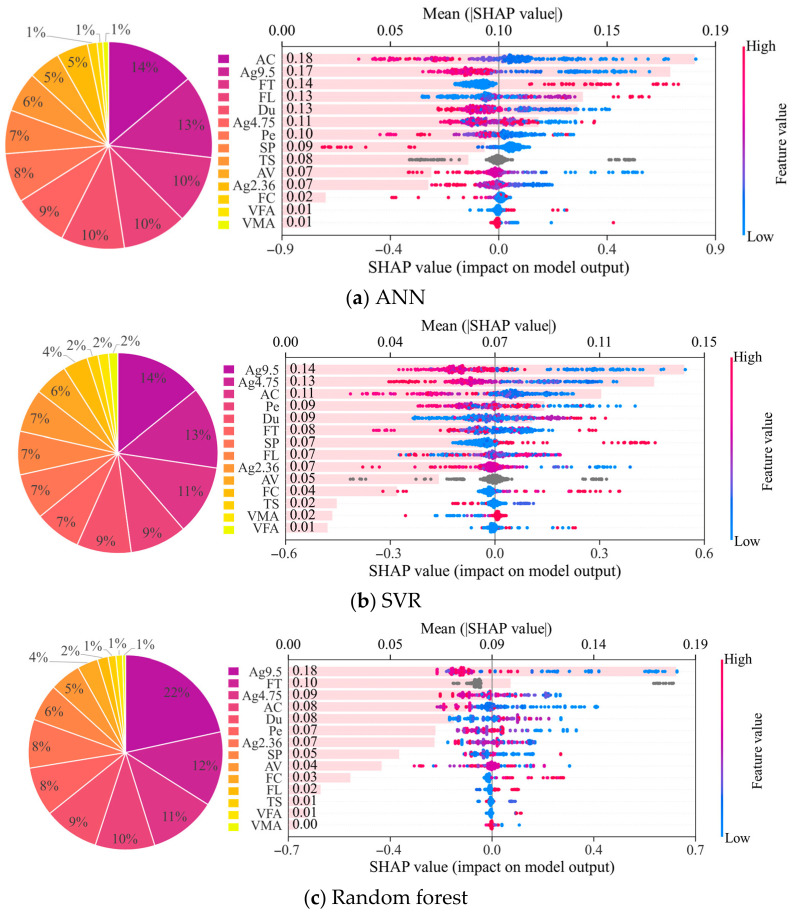

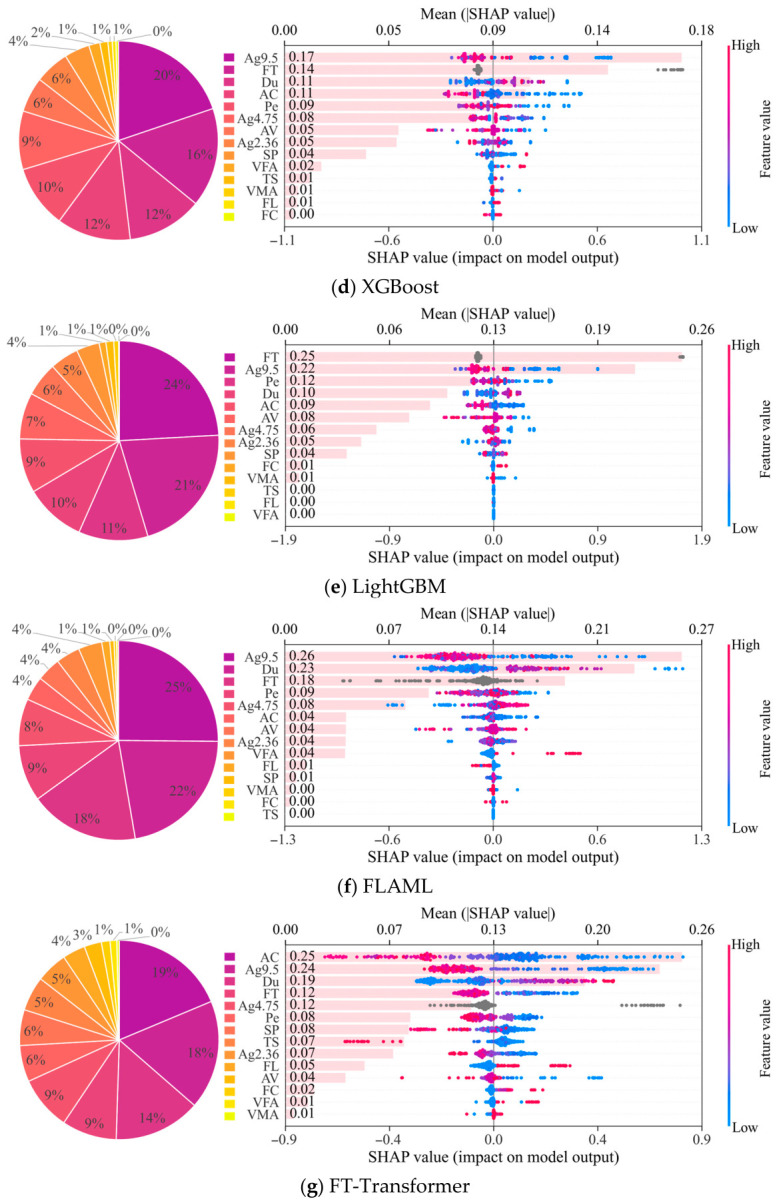
Summary plot of SHAP-based interpretation of the outputs from the remaining seven machine learning models: (**a**) ANN; (**b**) SVR; (**c**) RF; (**d**) XGBoost; (**e**) LightGBM; (**f**) FLAML; (**g**) FT-Transformer.

In addition to local explanation for a single sample, SHAP can also be used to quantify the overall influence of input variables on ST prediction across the full dataset. Figure 11 presents the global SHAP interpretation results of the TabPFN model, selected because of its superior predictive accuracy. The pie chart reports the proportional contribution of each feature based on the sum of absolute SHAP values, while the beeswarm plot further illustrates the distribution and polarity of feature effects for all samples. In the beeswarm plot, each point represents a sample, and the color scale indicates the feature value from low to high. The horizontal axis corresponds to the SHAP value, where positive and negative values denote increasing and decreasing effects on the predicted ST, respectively. Features are ordered by mean absolute SHAP value, allowing for direct comparison of their overall importance. The results show that Ag4.75 is the most influential predictor, with AC, Ag9.5, FT, Pe, and Du also contributing substantially, whereas VMA, FC, FL, and TS play relatively minor roles in the model output. The beeswarm plots of the remaining seven models are shown in Figure A2 of Appendix C.

As reflected by the pie charts in Figure 11 and Figure A2, the relative importance of individual variables is not identical across models; however, a clear overall pattern can still be observed. Based on the averaged percentages summarized in Table 5, Ag9.5, AC, Du, FT, and Ag4.75 are identified as high-impact variables, each contributing more than 10% on average. Pe, SP, Ag2.36 and AV fall into the medium-impact group, with average contributions between 5% and 10%. In contrast, FL, TS, FC, VFA, and VMA exhibit average contribution percentages below 5%, indicating relatively limited influence on the model output. In aggregate, high-, medium-, and low-impact features account for 61.1%, 30.6%, and 8.3% of the total influence, respectively, highlighting the dominant role of a small subset of variables in determining ST prediction.

#### 3.4.2. Feature-Wise Dependence Analysis

**Figure 12:**
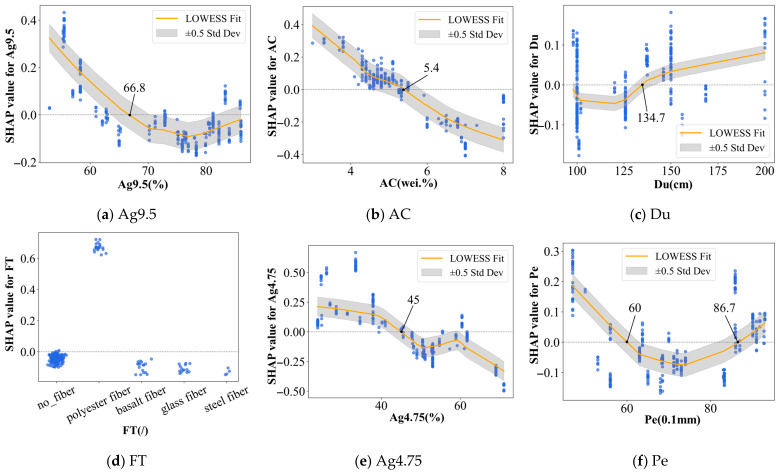

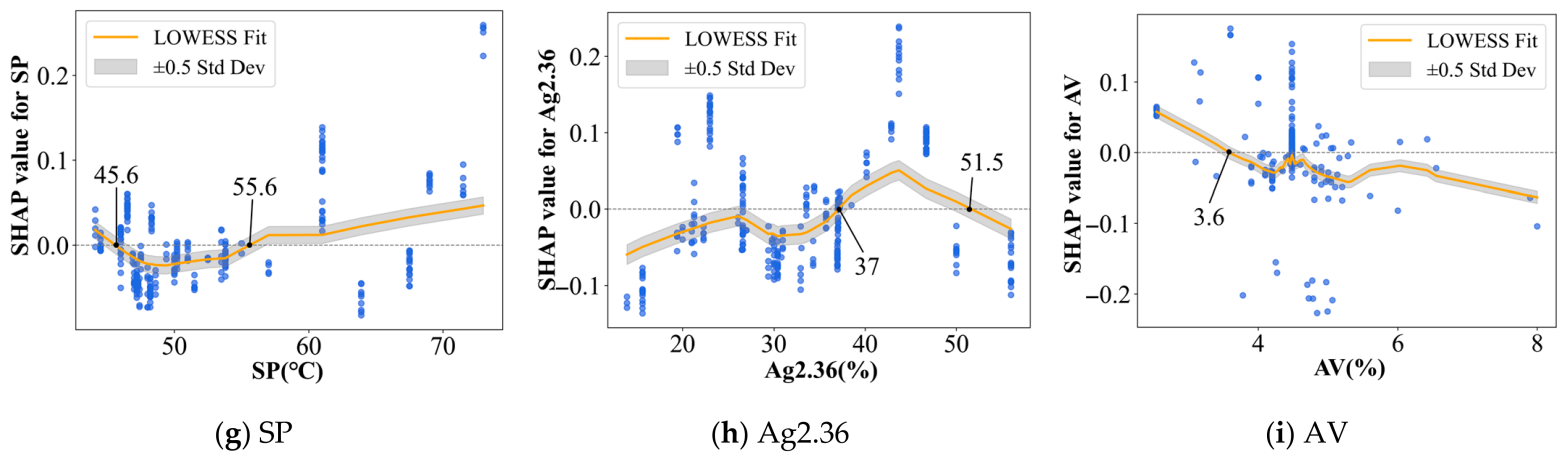
SHAP dependence plots of the nine key input variables for ST prediction based on the TabPFN model.

To examine the variation patterns and possible threshold behaviors of key variables affecting ST, Figure 12 displays the SHAP dependence plots of all input features obtained from the TabPFN model, thereby revealing how each parameter influences the ST of asphalt concrete.

Based on Table 5, the nine variables shown in Figure 12 (Ag9.5, AC, Du, FT, Ag4.75, Pe, SP, Ag2.36 and AV) were selected for dependence analysis because they comprise all high- and medium-impact features and jointly explain 91.7% of the total average SHAP contribution for ST prediction. Their SHAP dependence plots were fitted with LOWESS curves and accompanied by ±0.5 standard deviation error bands to characterize nonlinear trends and local uncertainty. This visualization facilitates parametric interpretation of the relationships between feature values and their corresponding SHAP effects on the predicted splitting strength. In contrast, the five low-impact variables (FL, TS, FC, VFA, and VMA), whose combined contribution is only 8.3%, were excluded from further discussion. Since FT is a categorical descriptor, its plot is presented as grouped scatter distributions for different fiber types rather than as a continuous fitted curve.

When the baseline ST value is 1.34 (see Figure 10a), the SHAP dependence plots in Figure 12 show that the nine dominant variables can be classified into three types according to their influence patterns. First, Ag9.5, AC, Ag4.75 and AV exhibit overall negative correlations with ST, as shown in Figure 12a,c,e,i, suggesting that larger values of these variables are generally unfavorable for improving splitting strength. From a physical perspective, this pattern can be attributed to the progressive weakening of the internal load-carrying structure of the mixture. Higher Ag9.5 and Ag4.75 passing rates generally indicate a gradation shift toward a less effective aggregate skeleton and weaker interlocking action [87]. Likewise, excessive AC may lead to an overly binder-rich system, in which thick asphalt films reduce the contribution of aggregate interlock to tensile resistance [88]. For AV, its negative effect is more direct, since a higher void content increases internal discontinuities and stress concentration, thus making crack initiation and propagation easier during the splitting process [89]. Second, Du shows an overall positive correlation with ST, as illustrated in Figure 12c, with its SHAP contribution becoming positive after a certain threshold. This trend is physically reasonable because higher ductility indicates a greater ability of the asphalt binder to accommodate tensile deformation and dissipate fracture energy, thereby reducing stress concentration and delaying crack propagation during the splitting process [90]. Third, Pe, Ag2.36, and SP display non-monotonic effects on ST, as shown in Figure 12f–h, indicating that their contributions vary across different value ranges. This non-monotonic behavior is physically plausible because Pe, Ag2.36, and SP affect ST through structural and binder property balance rather than through a simple linear mechanism, so only certain value ranges are favorable for resisting splitting failure [91,92,93]. For the categorical variable FT in Figure 12d, the SHAP distribution reveals a pronounced category-dependent pattern rather than a uniform fiber reinforcement effect. Polyester fiber is associated with the highest positive contribution, whereas the other fiber categories show lower or near-neutral SHAP values. Given the imbalance among fiber categories in the present database, these results should be interpreted as dataset-dependent relative model contributions rather than definitive judgments on the intrinsic effectiveness of individual fiber types. According to these relationships, the following thresholds or favorable ranges are suggested for enhancing ST performance: Ag9.5 < 66.8%, AC < 5.4 wt.%, Du > 134.7 cm, Ag4.75 < 45.0%, Pe < 60 or Pe > 86.7 (0.1 mm), SP < 45.6 °C or SP > 55.6 °C, 37.0% < Ag2.36 < 51.5% and AV < 3.6%.

## 4. Graphical User Interface Platform

**Figure 13:**
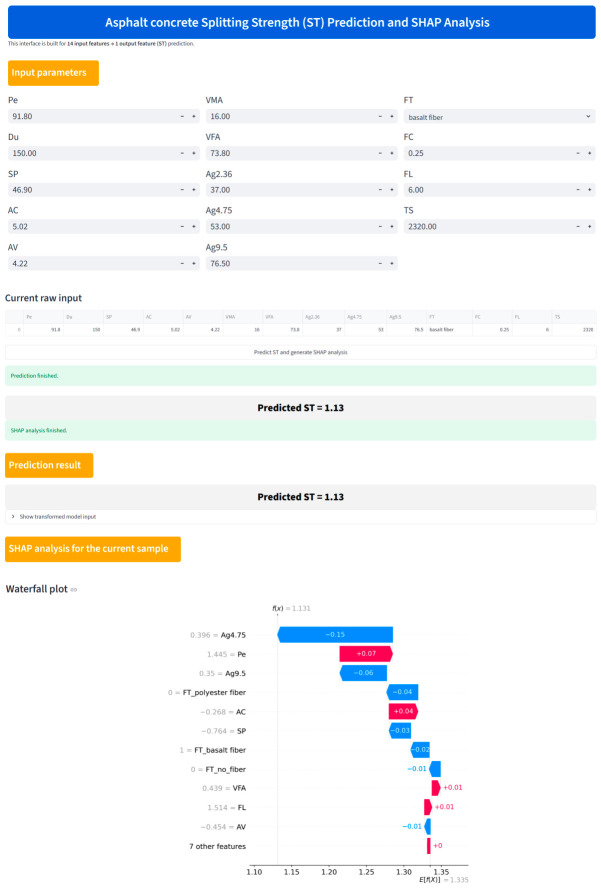
Graphical user interface for ST prediction, SHAP-based interpretation, and sample-level demonstration.

As shown in Figure 13, the graphical user interface (GUI) was developed in Python 3.12.3 based on the Streamlit framework. It allows users such as pavement engineers, laboratory researchers, and mixture designers to input 14 variables related to asphalt properties, aggregate gradation, and fiber characteristics for ST prediction. After entering the required parameters, the current raw input is displayed in tabular form, and the user can click the prediction button to obtain the predicted ST value generated by the deployed pretrained model. In addition, the GUI provides SHAP-based interpretability analysis for the current sample. A waterfall plot is displayed to show how individual input features contribute positively or negatively to the final ST prediction, thereby improving the transparency of prediction process. Therefore, the developed GUI serves not only as a practical prediction tool, but also as an interpretable decision support interface for asphalt concrete evaluation and mixture design.

To further validate the practical applicability of the GUI, a real sample from existing studies was used as a demonstration case [37], where the input values are Pe = 91.8 (0.1 mm), Du = 150 cm, SP = 46.9 °C, AC = 5.02 wt.%, AV = 4.22%, VMA = 16%, VFA = 73.8%, Ag2.36 = 37%, Ag4.75 = 53%, Ag9.5 = 76.5%, FT = Basalt fiber, FC = 0.25 wt.%, FL = 6 mm, and TS = 2320 MPa. For this sample, the actual ST is 1.14 MPa, while the GUI prediction of ST is 1.13 MPa, with a relative error of 0.88%. This result indicates that the deployed GUI is able to provide reliable sample-level prediction for real mixture data. Meanwhile, SHAP waterfall plot further explains the predicted result by identifying the main variables that increase or decrease ST for current sample. In this way, the GUI enables users not only to obtain a rapid ST estimate, but also to understand which mixture design variables are most effective for the prediction, thereby making the tool more useful for practical mixture screening and adjustment. The platform is available at https://st-gui-app-nlj7snzfjkvdaqfqf4yvkv.streamlit.app/ (access on 25 March 2026).

## 5. Limitations and Future Work

### 5.1. Overall Effectiveness

This study demonstrates the overall effectiveness of an interpretable data-driven framework for ST prediction of asphalt concrete by integrating dataset preprocessing, multi-model learning, hyperparameter optimization, SHAP-based explanation, and a user-oriented GUI platform. The results presented in Section 3 indicate that all eight machine learning models achieved acceptable prediction accuracy. Among them, TabPFN showed the strongest overall performance across the 20 Monte Carlo random splits and obtained the highest composite score, demonstrating that the proposed framework can effectively learn the complex nonlinear relationships between asphalt-, aggregate-, and fiber-related variables and the resulting splitting strength. Beyond predictive accuracy, the SHAP analysis further improves the engineering usefulness of the framework by identifying the dominant variables and clarifying their different influence patterns, including negative, positive, non-monotonic, and category-dependent effects. Therefore, the main value of the present work lies not only in accurate ST prediction, but also in providing interpretable parameter-level guidance for mixture design and offering a practical basis for future digital design tools for asphalt concrete.

### 5.2. Challenges and Limitations

Despite the promising results, several limitations remain. Since the database was assembled from different published articles, literature-derived data variability is unavoidable. In particular, differences in material sources, mix design details, specimen preparation procedures, testing conditions, and reporting practices may introduce additional uncertainty to dataset to affect the consistency, comparability, and transferability of developed models. Although data cleaning, variable unification, and repeated Monte Carlo random-splitting evaluation were adopted in this study to improve robustness, these measures cannot completely eliminate the inherent heterogeneity associated with cross-study data integration. Therefore, the predictive results and the corresponding interpretation outcomes should be understood in the context of a literature-derived dataset, and caution is still needed when extending the present conclusions to highly standardized or project-specific engineering conditions. In addition, the distribution of fiber types is not fully balanced, and this may reduce the reliability of the category-specific patterns identified for FT. It should therefore be emphasized that the SHAP results for FT reflect relative model contributions under the present dataset rather than definitive judgments on the intrinsic effectiveness of each fiber type. Moreover, although SHAP provides useful interpretability, it describes model-learned associations rather than causal mechanisms, meaning that the reported thresholds and favorable intervals should be treated as empirical guidance. Finally, the present framework is limited to ST prediction and has not yet been extended to long-term service behavior, environmental coupling effects, or full engineering life-cycle evaluation.

### 5.3. Opportunities for Future Research

Future studies should further strengthen the data basis and expand the application range of the current framework. An important direction for future work is to establish a larger and more standardized asphalt concrete database with more balanced fiber categories, more complete reporting of material properties, and more consistent testing protocols, so that model training and interpretation can become more robust and transferable. On this basis, future studies can extend the framework from single-property prediction to multi-objective design by jointly considering strength, cracking resistance, rutting resistance, durability, and sustainability indicators, thereby supporting more comprehensive optimization of asphalt mixtures. In parallel, combining SHAP with stronger causal analysis, uncertainty quantification, and external validation from laboratory or field data would make the design recommendations more credible and engineering-oriented. Finally, the current GUI can be further upgraded into a continuously updated intelligent platform in which new data are periodically incorporated, models are retrained, and users can obtain not only ST predictions but also dynamic recommendation ranges for mixture parameters under different engineering scenarios.

## 6. Conclusions

This study developed an interpretable ML framework to predict the splitting strength (ST) of asphalt concrete using a literature-based database with fourteen input variables associated with asphalt properties, aggregate gradation, and fiber characteristics. Eight machine learning models were developed and compared, and the framework further integrated hyperparameter optimization, SHAP-based interpretation, and a GUI platform to improve both predictive capability and engineering usability. Based on the findings of this study, the following conclusions can be drawn.

- All eight ML models demonstrated satisfactory capability for ST prediction, indicating that machine learning can effectively capture the nonlinear relationships between mixture design variables and splitting strength. Among them, TabPFN achieved the best overall performance over 20 Monte Carlo random splits, with the lowest average RMSE and MAE and the highest average R^2^. XGBoost, LightGBM, RF, and FLAML also showed relatively competitive performance, whereas SVR was slightly weaker but still acceptable. By contrast, FT-Transformer and ANN exhibited comparatively lower predictive capability. Overall, TabPFN was identified as the most effective model for ST prediction in the present dataset.
- The SHAP analysis showed that the prediction of ST is mainly governed by a limited number of dominant variables. Based on the average feature contributions across the eight models, Ag9.5, AC, Du, FT and Ag4.75 were identified as high-impact variables, while Pe, SP, Ag2.36, and AV were classified as medium-impact variables. Together, these nine variables accounted for 91.7% of the total average SHAP contribution, whereas FL, FC, TS, VFA, and VMA had relatively minor influence. In addition, the SHAP force plot analysis for a representative sample showed that TabPFN provided the closest prediction to the actual ST value, further confirming its strong local interpretability and predictive reliability.
- The SHAP dependence analysis further revealed that the dominant variables exhibit varying influence patterns on ST, including overall negative correlations, positive correlations, non-monotonic effects, and category-dependent effects. Specifically, Ag9.5, Ag4.75, AC, and AV showed overall negative correlations with ST; Du showed an overall positive correlation; and Pe, Ag2.36, and SP exhibited non-monotonic relationships. For the categorical feature FT, polyester fiber showed a comparatively stronger positive contribution to ST than the other fiber types in the present dataset under the current data conditions. Based on the dependence analysis, the favorable ranges for improving ST were identified as Ag9.5 < 66.8%, Ag4.75 < 45.0%, AC < 5.4 wt.%, AV < 3.6%, Du > 134.7 cm, Pe < 60 or > 86.7 (0.1 mm), 37.0% < Ag2.36 < 51.5%, and SP < 45.6 °C or > 55.6 °C.
- Beyond model construction and interpretation, this study also developed a GUI platform to enhance the accessibility and applicability of the developed framework. By integrating prediction and SHAP-based explanation into a user-oriented interface, the platform provides a practical tool for estimating ST and understanding the role of individual design variables. Overall, the proposed framework offers not only accurate prediction of splitting strength, but also interpretable guidance for mixture design, thereby demonstrating the potential of explainable artificial intelligence in the intelligent design and optimization of asphalt concrete.

## Figures and Tables

**Table 1:** Recent studies (2022–2025) on ML-based prediction of asphalt mixture properties.

Approaches	Prediction Target	Limitations	Ref.
Statistical and ML regression models with hyperparameter optimization and SHAP interpretation for hot-mix asphalt	Dynamic modulus	Focused on dynamic modulus rather than splitting strength; no GUI/tool deployment; newer and potentially more powerful models were not examined.	[25]
Explainable machine learning models for asphalt pavements	Marshall stability and flow	Focused on Marshall-related indicators; limited emphasis on splitting strength and sample-level deployment tools	[26]
BPNN and SVM under interactive loading condition analysis	Asphalt mixture strength under interactive conditions	Mainly based on conventional ML models; no SHAP-based global/local interpretation framework; no interactive application platform	[27]
ML-based application for Oklahoma plant-produced mixes	Cracking Tolerance Index (CTIndex)	Targeted cracking resistance rather than splitting strength; application-oriented, but not coupled with a systematic multi-model interpretable framework	[28]
Regression-based ML with CTGAN data augmentation and SHAP	Marshall stability and flow	Target remained Marshall design indicators; reliance on synthetic data augmentation; no comparison with recent tabular foundation models	[29]
Hybrid deep neural network and ensemble learning	Marshall stability	Focused on single Marshall-related target; limited interpretability and no GUI deployment	[30]
Attention-based tabular network + ensemble learning + GAN	Stiffness modulus	Addressed stiffness modulus instead of splitting strength; more emphasis on predictive architecture than on interpretability and engineering usability	[31]

**Table 2:** Overview of fiber types and their amounts.

Fiber Types	Sample Size
Basalt fiber	17
Glass fiber	14
Polyester fiber	20
Steel fiber	4
No fiber	241

**Table 3:** Distribution of fourteen input variables and the output variable.

Variable	Unit	Min	Q1	Q2	Q3	Max	Mean	STD
Pe	0.1 mm	47	63	71.2	85.9	93	71.65	14.05
Du	cm	98	100	101	150	200	125.65	31.21
SP	°C	44.1	47.2	50	57	73	53	7.87
AC	wt. %	3	4.6	4.9	6.5	8	5.35	1.17
Ag2.36	%	13.9	26.58	32.92	40.15	56	33.54	10.9
Ag4.75	%	23.9	37.9	50.77	58.89	71	47.66	13.53
Ag9.5	%	53	62.76	76.16	81.2	86	72.88	10.3
AV	%	2.54	4.01	4.34	4.95	8	4.41	0.98
VMA	%	12.1	14.94	15.36	16.2	65.6	17.08	8.65
VFA	%	17.11	69.03	72.95	82.59	83.41	72.91	11.81
FC	%	0	0	0	0	3	0.09	0.36
FT	/	/	/	/	/	/	/	/
TS	MPa	0	0	0	0	3250	237.17	735.13
FL	mm	0	0	0	0	12	1.23	2.94
ST	MPa	0.13	0.71	1.1	1.48	5.15	1.33	0.91

**Table 4:** Overview of machine learning models.

No.	Model	Category	Notes
1	TabPFN	Foundation model	Transformer-based prediction
2	ANN	Classical	Nonlinear regression
3	SVR	Classical	Kernel-based regression
4	RF	Ensemble—Bagging	Bagging of decision trees
5	XGBoost	Ensemble—Boosting	Boosting model with regularization
6	LightGBM	Ensemble—Boosting	Efficient histogram-based gradient boosting
7	FLAML	AutoML	Automated model selection and hyperparameter optimization
8	FT-Transformer	Transformer-based deep learning	Attention-based tabular regression

**Table 5:** Ranking of variable importance for ST prediction.

Variables	Mean Contribution (%)	Impact Level
Ag9.5	18	High
AC	11.7	High
Du	10.7	High
FT	10.4	High
Ag4.75	10.3	High
Pe	8.2	Medium
SP	6.7	Medium
Ag2.36	6.3	Medium
AV	5.2	Medium
FL	4.2	Low
TS	3.5	Low
FC	2.1	Low
VFA	1.8	Low
VMA	0.9	Low

## Data Availability

The original contributions presented in the study are included in the article, further inquiries can be directed to the corresponding authors.

## Abbreviations

**Abbreviation**	**Full Name**
AC	Asphalt content
Ag2.36	2.36 mm aggregate passing rate
Ag4.75	4.75 mm aggregate passing rate
Ag9.5	9.5 mm aggregate passing rate
AV	Air voids
Du	Ductility
FC	Fiber content
FL	Fiber length
FT	Fiber type
Pe	Penetration
SP	Softening point
ST	Splitting strength
TS	Tensile strength
VFA	Voids filled with asphalt
VMA	Voids in mineral aggregate

## Appendix A. Data Description

**Table A1:** Software environment and main libraries used in this study.

Category	Software/Library	Version
Programming environment	Python	3.13.12
Numerical computing	NumPy	2.4.3
Data processing	pandas	3.0.1
Machine learning	scikit-learn	1.8.0
Machine learning	XGBoost	3.2.0
Machine learning	FLAML	2.5.0
Machine learning	PyTorch-Tabular	1.1.0
Machine learning	LightGBM	4.6.0
Foundation model	TabPFN	6.4.1
Explainable AI	SHAP	0.51.0
Optimization	pymoo	0.6.1.6
Visualization	matplotlib	3.10.8
Visualization	seaborn	0.13.2
Model persistence	joblib	1.5.3
GUI development	Streamlit	1.55.0

## Appendix B. Model Configuration and Performance Evaluation

**Table A2:** Hyperparameter combinations adopted for each model.

Models	Hyperparameters
TabPFN	default hyperparameters; random_state = 42
ANN	hidden_layer_sizes = (64,); alpha = 8.14e−06; learning_rate_init = 0.01; batch_size = 60; max_iter = 225; activation = relu; solver = adam; random_state = 42; early_stopping = True; validation_fraction = 0.10
SVR	kernel = rbf; C = 6.02; epsilon = 0.08; gamma = 0.07
RF	n_estimators = 370; max_depth = 13; min_samples_split = 2; min_samples_leaf = 1; max_features = log2; bootstrap = True; random_state = 42; n_jobs = −1
XGBoost	n_estimators = 207; max_depth = 9; learning_rate = 0.03; subsample = 0.63; colsample_bytree = 0.60; objective = reg:squarederror; tree_method = hist; random_state = 42; n_jobs = −1
LightGBM	n_estimators = 495; max_depth = 8; learning_rate = 0.05; subsample = 0.80; colsample_bytree = 0.83; num_leaves = 46; min_child_samples = 12; reg_alpha = 0.11; reg_lambda = 0.09; random_state = 42; n_jobs = −1; verbosity = −1
FLAML	task = regression; metric = rmse; time_budget = 60 s; seed = 42; verbose = 0
FT-Transformer	valid_size = 0.20; max_epochs = 100; batch_size = 64; auto_lr_find = False; checkpoint_mode = min; early_stopping_patience = 10; seed = 42

**Table A3:** Average testing performance of the models for ST prediction over 20 Monte Carlo random splits.

Model	Metrics				
	RMSE	MAE	MAPE	MAD	R^2^
TabPFN	0.34 ± 0.10	0.21 ± 0.02	21.69 ± 3.29	0.14 ± 0.03	0.85 ± 0.08
ANN	0.66 ± 0.50	0.35 ± 0.13	36.78 ± 12.49	0.21 ± 0.08	0.23 ± 1.31
SVR	0.37 ± 0.11	0.24 ± 0.04	25.18 ± 5.47	0.16 ± 0.04	0.82 ± 0.08
RF	0.37 ± 0.10	0.23 ± 0.03	23.75 ± 3.36	0.14 ± 0.02	0.82 ± 0.09
XGBoost	0.36 ± 0.10	0.22 ± 0.02	22.67 ± 3.09	0.13 ± 0.02	0.83 ± 0.09
LightGBM	0.36 ± 0.10	0.23 ± 0.03	22.97 ± 3.34	0.14 ± 0.02	0.83 ± 0.09
FLAML	0.36 ± 0.10	0.22 ± 0.02	22.74 ± 3.10	0.13 ± 0.02	0.83 ± 0.09
FT-Transformer	0.46 ± 0.14	0.30 ± 0.04	31.00 ± 5.82	0.19 ± 0.05	0.71 ± 0.18

Note: For the 20 Monte Carlo random splits, the random seed was set as seed = 42 + split_id.

**Table A4:** Paired *t*-test results of RMSE between TabPFN and the other models over 20 Monte Carlo random splits.

Comparison Model	TabPFN Mean RMSE	Comparison Mean RMSE	t-Statistic	Adjusted *p*-Value	Significance
FT-Transformer	0.335	0.457	−10.257	2.44 × 10^−8^	Yes
RF	0.335	0.366	−5.599	1.28 × 10^−4^	Yes
LightGBM	0.335	0.358	−4.589	1.00 × 10^−3^	Yes
FLAML	0.335	0.36	−4.46	1.07 × 10^−3^	Yes
XGBoost	0.335	0.356	−3.746	4.10 × 10^−3^	Yes
SVR	0.335	0.368	−3.128	1.11 × 10^−2^	Yes
ANN	0.335	0.655	−2.864	1.11 × 10^−2^	Yes

Note: Paired *t*-tests were performed based on the results of 20 Monte Carlo random splits. Adjusted *p*-Values were obtained using the Holm correction for multiple comparisons.

**Table A5:** Paired *t*-test results of R^2^ between TabPFN and the other models over 20 Monte Carlo random splits.

Comparison Model	TabPFN Mean R^2^	Comparison Mean R^2^	t-Statistic	Adjusted *p*-Value	Significance
FT-Transformer	0.853	0.727	7.067	7.03 × 10^−6^	Yes
RF	0.853	0.824	4.654	1.04 × 10^−3^	Yes
FLAML	0.853	0.832	4.078	3.20 × 10^−3^	Yes
LightGBM	0.853	0.83	3.264	1.63 × 10^−2^	Yes
XGBoost	0.853	0.832	2.975	2.33 × 10^−2^	Yes
SVR	0.853	0.825	2.681	2.96 × 10^−2^	Yes
ANN	0.853	0.225	2.141	4.55 × 10^−2^	Yes

Note: Paired *t*-tests were performed based on the results of 20 Monte Carlo random splits. Adjusted *p*-values were obtained using the Holm correction for multiple comparisons.
